# Bleb geometry and morphology after Preserflo Microshunt surgery: Risk factors for surgical failure

**DOI:** 10.1371/journal.pone.0286884

**Published:** 2023-06-08

**Authors:** Marta Ibarz Barberá, Jose Luis Hernández-Verdejo, Jean Bragard, Laura Morales-Fernández, Lola Rodríguez-Carrillo, Fátima Martínez Galdón, Pedro Tañá, Miguel A. Teus

**Affiliations:** 1 Oftalvist Group, Moncloa HLA Hospital, Madrid, Spain; 2 Complutense University of Madrid, Clinical and Experimental Eye Research, Madrid, Spain; 3 University of Navarra, Pamplona, Spain; 4 Clínico San Carlos Hospital, Madrid, Spain; 5 Oftalvist Group, Vistahermosa Hospital, Alicante, Spain; 6 Príncipe de Asturias University Hospital, University of Alcalá, Alcalá de Henares, Madrid, Spain; Pusat Perubatan Universiti Kebangsaan Malaysia, MALAYSIA

## Abstract

**Purpose:**

To investigate the possible risk factors for treatment failure in patients who had undergone Preserflo Microshunt (PMS) implantation, using anterior-segment optical coherence tomography (AS-OCT) to analyze the internal structures of the bleb.

**Methods:**

The PMS blebs of 54 patients were evaluated with AS-OCT. A mathematical model was used to calculate the total filtering surface of the episcleral fluid cavity (EFC) and the hydraulic conductivity (HC) of the bleb wall. Complete and qualified success were defined as IOP between 6 and 17 mmHg with or without glaucoma medication. The relation between baseline characteristics and probability of bleb success was analyzed by bivariate and multivariate logistic regression. The main outcome measures were mean bleb wall thickness (BWT), reflectivity (BWR), HC, mean horizontal and vertical diameter and total filtering surface (TFS) of the EFC.

**Results:**

Blebs from 74% patients were considered as complete success and 26% as failure. BWR and BWT increased linearly up to the first year in both groups. BWR was higher in the group failure (p = 0.02) and BWT in the group success (p<0.001). EFC was wider and shorter in the success group (p = 0.009, p = 0.03). Higher TFS showed a negative correlation with IOP (r = -0.4, p = 0.002). Higher baseline IOP was associated with success of PMS by multivariate analysis (p = 0.01). Mean HC, 0.034 ± 0.008 (μL/min)/mm^2^/mmHg, was negatively correlated with bleb surface (r = -0.5, p<0.0001) and wall´s thickness (r = -0.3, p = 0.01).

**Conclusions:**

AS-OCT revealed that successful PMS blebs could show either thick hyporreflective walls or wide filtering surfaces with thin capsules. A higher baseline IOP increased the probability of surgical success.

## Introduction

The Preserflo MicroShunt (Santen Pharmaceutical Company, Ltd., Osaka, Japan) belongs to a group of relatively new microtube shunts without an end plate designed to restrict the outflow due to a reduction in the luminal diameter [1]. The pressure differential or pressure drop between both ends of the tube (P) determines the volume of aqueous humor that is transferred to the subconjunctival space. The higher the pressure drop is, the lower the volume of aqueous humor (AH) that the tube delivers to create a long-term functioning bleb [2].

The pressure drop through the PMS (P = 2.6 mmHg)^3^ has been increased by 3 orders of magnitude in comparison to that with traditional tube shunts (P = 0.008 mmHg) [3] to control the outflow without the need for a valved mechanism or an external ligature to avoid hypotony. With the PMS, the AH and its cytokines [4] contact the sub-Tenon´s space from the immediate postoperative period into a space created by the surgeon´s dissection of the conjunctiva. There is no plate at the end of the tube to separate the tissues, prevent them from sticking to each other and enlarge the filtering surface, the PMS bleb is supported only by the hydrostatic pressure of the AH. Fibrosis, the main cause of bleb failure, needs to be reduced with this device by the use of adjunctive mitomycin C (MMC) [5], even though the tube´s material (polystyrene‐block‐isobutylene‐block‐styrene, SIBS) has been shown to reduce the inflammatory response [6, 7].

AS-OCT analysis of the bleb morphology of PMSs has revealed the presence of AH in a deep location beneath Tenon´s and above the episclera 3 months after surgery [8]. The subconjunctival space showed multiple layers of tissue separated by liquid and the presence of AH inside an episcleral fluid cavity, both of which are prognostic factors for success after trabeculectomy (TB) [2]. We hypothesized that early PMS bleb morphology could determine long-term function and that long-term function could be related to qualitative and quantitative features of the internal structure of the bleb. In addition, the analysis of the bleb morphology and geometry could help elucidate the mechanism of action of this particular SIBS tube without an end-plate. In order to do so, we visualized the internal structure of the PMS bleb with AS-OCT, calculated the filtering surface and hydraulic conductivity of the bleb wall with a geometrical model and evaluated the relationship of bleb parameters and IOP with surgical success.

## Materials and methods

This prospective interventional study was approved by the institutional review board of the Oftalvist Group (Madrid, Spain). The study design followed the tenets of the Declaration of Helsinki for biomedical research. All patients signed the informed consent previous to surgery and collection of data. Patients who met the eligibility criteria were enrolled between October 2019 and May 2021 at the Department of Glaucoma at the HLA Moncloa Hospital, Oftalvist Group, Madrid, Spain. A total of 54 patients with open angle glaucoma that were implanted with a PMS and fulfilled a one-year follow up period were included in the study. Inclusion criteria were as follows: best-corrected visual acuity (BCVA) of 20/200 or better; uncontrolled glaucoma under maximum tolerated medication; IOP ranging from 12 to 45 mmHg; phakic or pseudophakic patients treated with intracapsular lens implantation; individuals who showed rapid and significant loss of visual function [visual function index (VFI), mean deviation (MD) and glaucoma progression analysis (GPA) with the Humphrey Visual Field Analyzer, Carl Zeiss AG, Germany]. Both eyes could be included with a one-month interval between surgeries for uncomplicated cases. Although patients with previous failed minimally invasive glaucoma surgery (MIGS) can be recruited, none of them had other types of MIGS implantation before their inclusion in the study. Exclusion criteria were angle closure, congenital and neovascular glaucoma. Eyes with a history of previous filtering surgery (TB) were included when no sign of filtration was present at the slit lamp (flat, vascularized bleb) and by AS-OCT (uniform hyperreflective stroma, absence of microcysts and EFCs). In these cases, the PMS was implanted avoiding the previous filtration site. PMSs located inferiorly were also excluded from the study. Three patients were excluded from the study due to loss of follow up before the first year.

Preoperative baseline characteristics included sex, age, slit-lamp biomicroscopy, number of glaucoma medications, IOP measurement using Goldmann applanation tonometry, gonioscopy, central corneal thickness using ultrasound pachymetry (Topcon specular microscope SP-1P, Topcon Corporation, Tokyo, Japan), dilated fundus examination, visual field analysis (Humphrey Visual Analyzer, Carl Zeiss Meditec, Dublin, CA), visual acuity (Snellen decimal best corrected visual acuity), glaucoma type, previous glaucoma surgery (type and number) and lens status.

Postoperative evaluation was performed at 24 hours, one week, one month, 3 and 6 months and one year. Data included in the study from bleb evaluation was collected at one month, 6 months and one year and will be explained in a separate section. IOP was measured at every visit and the number of glaucoma medications was collected at 6 and 12 months. The mean number of needling and/or surgical revisions were reported by the end of the follow-up period (one year).

Definition of surgical success: IOP from 6 to 17 mmHg and ≥ 20% reduction from baseline, without medication (complete success) or with medication (qualified success). Loss of light perception or any other serious adverse event (blebitis, endophthalmitis…) was considered complete failure and the patient was withdrawn from the study. Surgical revision or needling were not considered as a reason to be withdrawn from the study. All surgical revisions were performed with the use of a biodegradable collagen matrix (Ologen, Aeon Astron Corporation, Taipei, Taiwan) and mitomycin C 0.2 mg/ml.

### Preserflo Microshunt implantation

The surgical technique has been previously reported by our group at [9]: https://journals.lww.com/glaucomajournal/Fulltext/2021/10000/Changes_to_Corneal_Topography_and_Biometrics_After.8.aspx.

### Anterior-segment optical coherence tomography

AS-OCT was performed in all patients using the “Cross Line” imaging method included in the software of the Avanti Widefield OCT (Optovue, Inc., Fremont, CA, USA).

#### Qualitative AS-OCT bleb analysis (Fig 1)

*Bleb wall´s reflectivity*: Multilayer: “High”, “medium”, “low” and “very low”. *Microcysts*: “none or few” vs. “many”. *Type of EFC*: Grades 0, 1, 2, 3 and 4.

**Fig 1:**
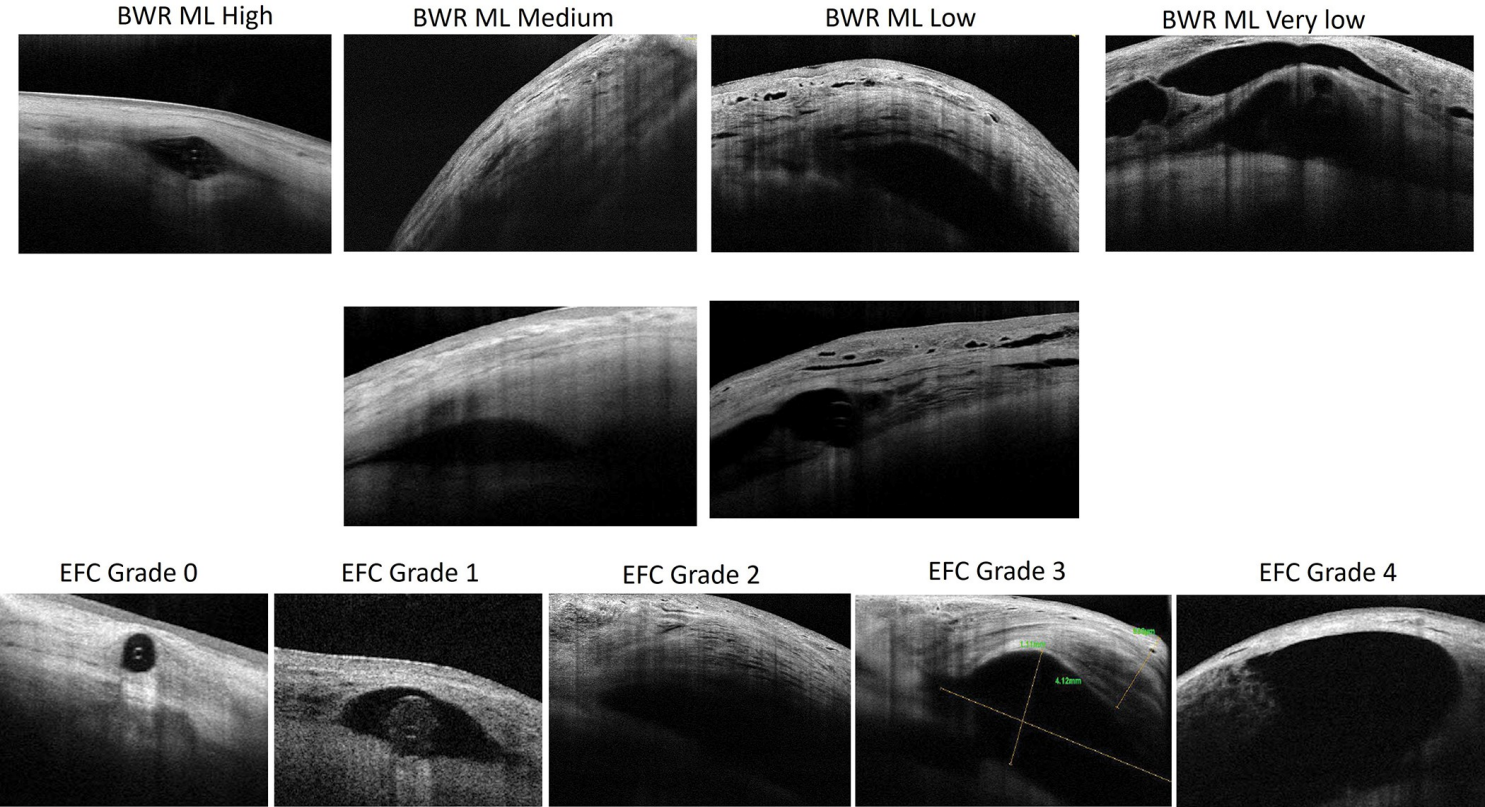
Qualitative classification of the bleb wall´s reflectivity (BWR) and episcleral fluid cavity (EFC). Multilayer (ML): High, medium, low, very low. Microcysts (MC): None or low vs. high; EFC: Grade 0: no aqueous, Grade 1: scarce liquid around the tube, Grade 2: discrete collection of aqueous, thick hyporeflective wall, Grade 3: large fluid cavity, mixed bleb wall (thin-hypereflective, thick-hyporeflective), Grade 4: Very large fluid cavity, thin bleb wall.

#### Quantitative AS-OCT bleb analysis (Fig 2), flow rate (Q) and hydraulic conductivity

**Fig 2:**
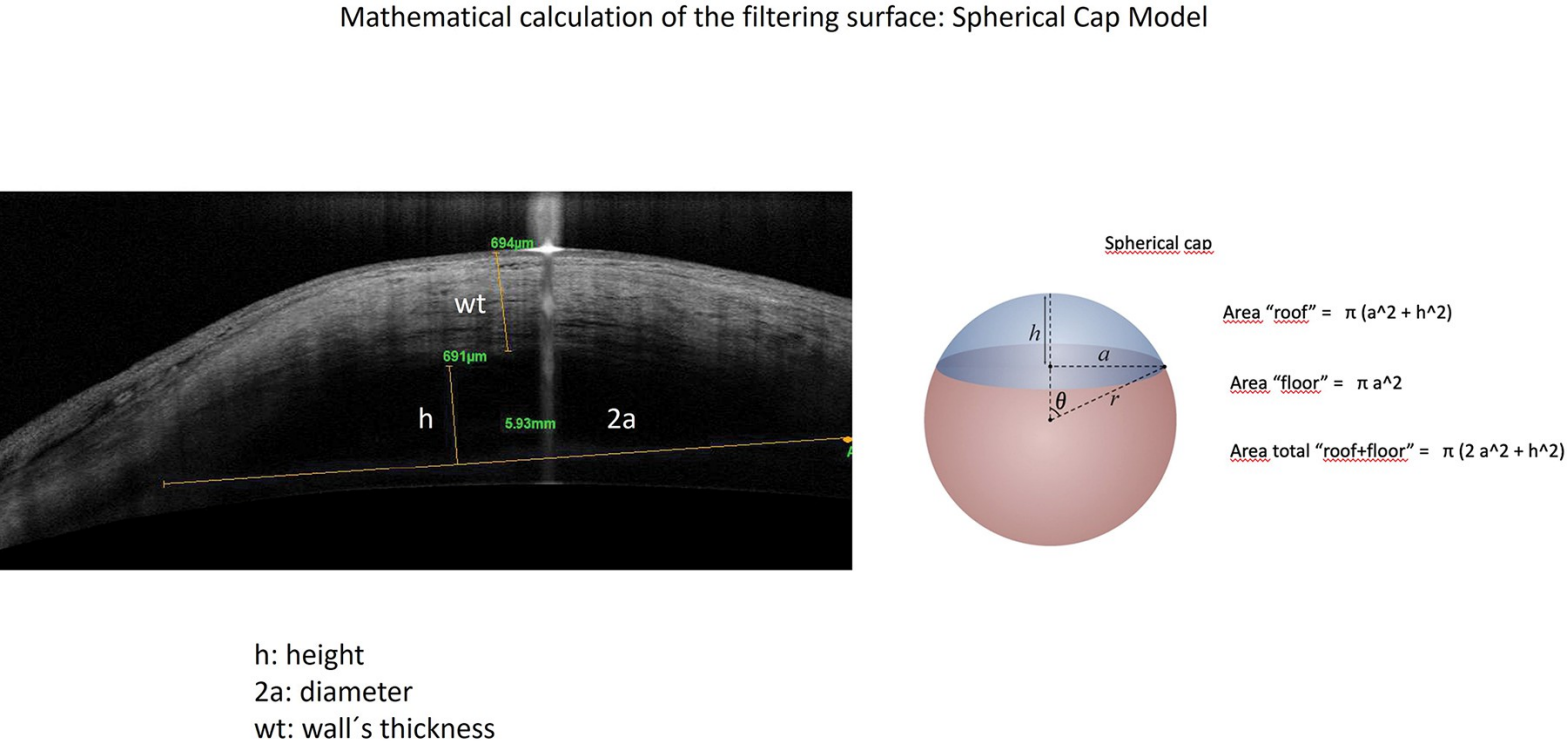
AS-OCT measurement of the EFC dimensions (height and width) and bleb wall´s thickness. Height (h): measured from the highest point of the EFC; “a”: radius; “2a” diameter (angle to angle of the EFC). Wall´s thickness (wt): Measured from the top of the EFC where the bleb wall´s thickness was considered maximum; “Area roof”: Tenon´s filtering surface: “Area floor” episcleral filtering surface. “Total filtering surface”: area roof + area floor.

The HC is a physical characteristic of the bleb wall, defined by Parker [10] as the flow rate (Q, μL/min) per unit membrane (mm^2^) at a unit pressure gradient (mmHg). The flow rate through PMS (Q) has been shown to be a linear function of pressure [3] and can be calculated for any IOP according to the Hagen Poiseuille´s law [1]. *Tubular flow (Q) and IOP* [3]: Flow through PMS (Q) expressed in g/(24 h):Q = 50.72 95% ic [50.71; 50.74] g/(24h); Q expressed in μL/min for an IOP 58.92 mmHg: Q = 35,22 (μL/min) at an IOP = 58.92 mmHg; Q can be calculated by a simple rule of three for every IOP: IOP 15 mmHg: Q = 8,9 μL/min. *Calculation of the HC of the bleb wall*: At 6 months and 1 year (once the maturation process of the bleb was completed), the flow though PMS (Q) was calculated using the IOP of each patient as explained above. The calculation of Q was necessary to calculate the HC, as well as the calculation of the total filtering surface with the “spherical cap” formula, detailed above (Fig 2). HC = Q (μL/min) / total filtering surface (mm^2^) / IOP (mmHg).

### Statistical analysis

#### Bivariate analysis

A cross-sectional analysis was performed to analyze the relation between a total of 15 independent variables and two dichotomic variables named “complete success” and “qualified success”. The independent nominal variables were sex, laterality, pseudoexfoliation, combined phaco-PMS surgery, and bleb wall´s reflectivity (microcysts). The ordinal variables were bleb wall´s reflectivity (multilayer) and EFC. Numerical variables were number of preoperative and postoperative glaucoma medications, baseline IOP, IOP at one week, 1 month, 6 months and one year, bleb wall´s thickness, EFC diameter and height, total height (EFC height + bleb wall´s thickness), total filtering surface and HC of the bleb. Nominal variables were analyzed by the Chi Squared test and Fisher´s exact test, while ordinal and numerical variables were analyzed by paired sample t test and Wilcoxon signed-rank test when the variables did not follow a normal distribution. The SAS software, version 9.4 (SAS Institute, Inc., Cary, NC, USA) was used for the analysis. Boxplots, bar graphs and dot plots from the univariate analysis of the variables was performed with Stata 17^®^ (StataCorp LLC, Texas, USA).

#### Multivariate logistic regression (LR) analysis

**Fig 3:**
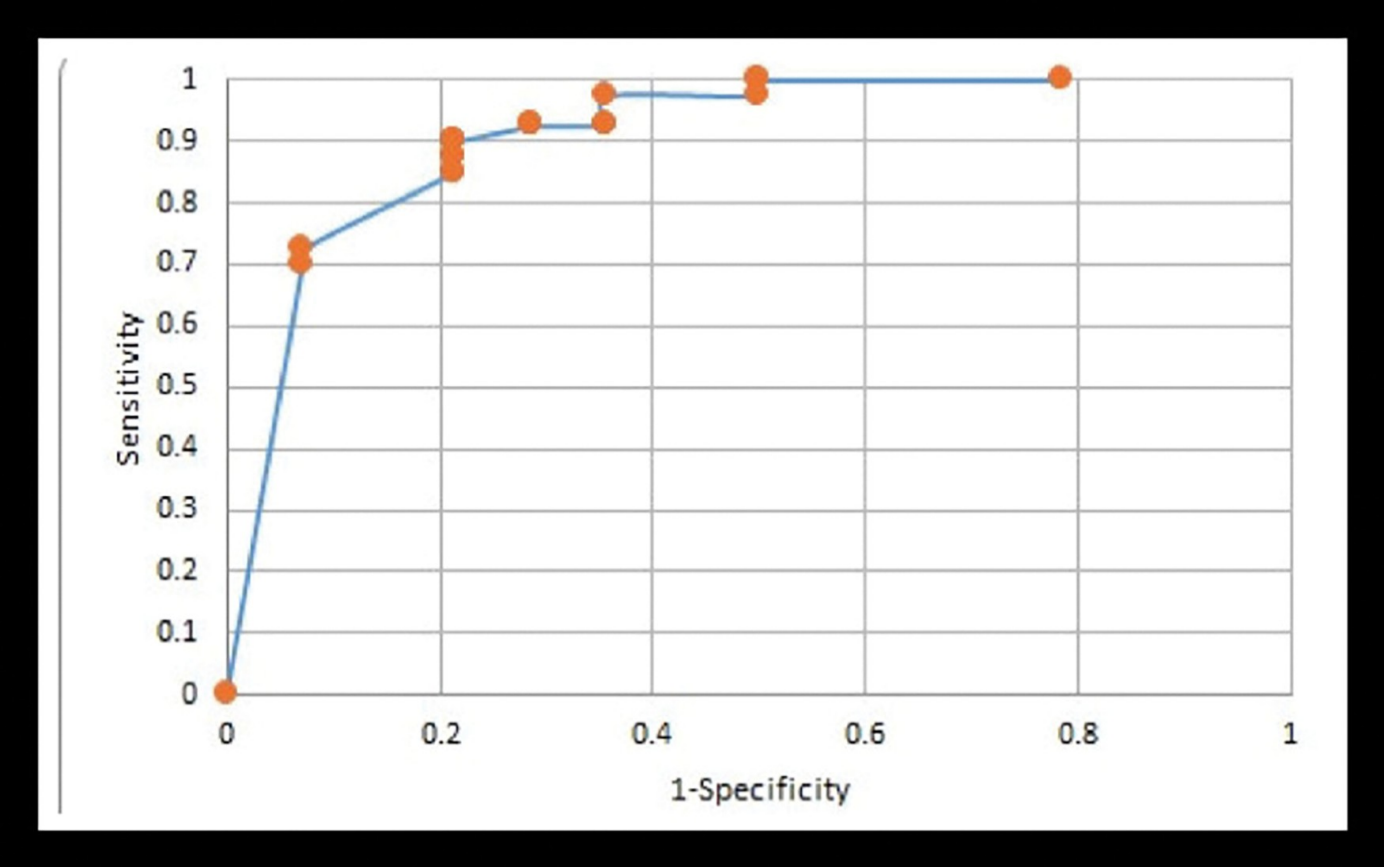
Receiver operating characteristic (ROC) curve for the multivariate logistic regression analysis. AUC: Area under the curve; Sensitivity, proportion of responders which are correctly identified; Specificity, proportion of non-responders which are correctly identified. Variables included in the model: Basal IOP, postoperative IOP, number of hypotensive medications, bleb wall´s thickness, EFC height, diameter and total filtering surface, total height of the bleb (EFC height + bleb wall´s thickness), All the postoperative variables were included at 1 week, 1 month, 6 months and 1 year). Only the independent variables, higher baseline IOP and lower IOP at one year, were significant predictive factors of complete surgical success one year after surgery. (ODDs ratio = 1.68, AUC of the ROC curve > 0.95).

All the variables included in the multivariate analysis that were statistically significant based on a p < 0.05 level were included in the Logistic Regression Model to assess the prognostic factors for bleb success. The model used the stepwise method. Inclusion criteria was set at α ≤ 0.15 and elimination criteria was β ≥ 0.05. The analysis was performed 6 and 12 months after surgery. The SAS software mentioned previously for the bivariate analysis was used to build the logistic regression model. The variables included in the model are available at Fig 3.

## Results

The PMS was implanted in 54 eyes of 51 patients who were followed up for one year. Baseline characteristics of the study population, divided by the main groups of overall success versus failure, are shown in Table 1.

**Table 1:** Baseline characteristics of the patient population.

	Total (n = 54)	Overall success (n = 44)	Complete success (n = 40)	Qualified success (n = 4)	Failure (n = 10)	P value
Mean age, yrs ± SD	73.1 ± 1.2	72.6 ± 9.4	72.3 ± 9.7	76 ± 4.8	75.3 ± 8.8	0.41
Male sex, % (n)	53.7 (29)	44.4 (24)	52.5 (21)	75 (3)	50 (5)	0.66
Laterality, % (n) LE	44.4 (24)	33.3 (18)	31.4 (17)	1.8 (1)	11.1 (6)	0.43
Central corneal thickness (μm, ± SD)	509.5 ± 4.1	509.8 ± 31	508.6 ± 31.1	521.5 ± 32	508.5± 28.5	0.94
Mean number of glaucoma medications, ± SD	2.7 ± 0.7	2.6 ± 0.7	2.6 ± 0.7	3	2.7 ± 0.6	0.75
Non combined surgery, % (n)	77.7 (42)	64.8 (35)	57.4 (31)	7.4 (4)	12.9 (7)	0.47
Combined surgery, % (n)	22.2 (12)	16.6 (9)	16.6 (9)	0	5.5 (3)	0.47
MD (dB, ± SD)	-13.5 ± 1.2	-13.1 ± 8.7	-13.7 ± 8.9	-8.2 ± 4.1	-14.8 ± 9.6	0.34
VFI (%,± SD)	62.2 ± 3.9	63 ± 27.6	61.6 ± 28.7	74.5 ± 10.4	59.5 ± 28.5	0.20
Baseline IOP (mmHg, ± SD)	21.1 ± 6.5	22.1 ± 6.8	22.3 ± 6.9	19.5 ± 5.8	16.8 ± 2	<0.01

The patient population was divided by groups of complete success, qualified success and failure 1 year after surgery. IOP: Intraocular pressure; MD: Mean deviation; SD: Standard Deviation; VFI: Visual Field Index.

No statistically significant differences were found between groups except for the baseline IOP, that was significantly higher in the complete success group (22.1 ± 6.8 vs. 16.8 ± 2 mmHg, p < 0.01). The groups of combined vs. non-combined surgery showed comparable rates of complete success (p = 0.93). The main type of glaucoma in the study population was primary open angle glaucoma (POAG, 51 eyes), 3 patients had pseudoexfoliative glaucoma and were analyzed together with POAG patients.

### Intraocular pressure and IOP-lowering medications

Mean (± SD) IOP (mmHg) decreased significantly from 21.1 ± 6.5 at baseline to 12.03 ± 0.4 at the final follow up visit (p<0.001). Similarly, the number of IOP-lowering medications decreased significantly from 2.7 ± 0.7 at baseline to 0.18 ± 0.5 at 1 year (p<0.001). Compared to baseline values, the overall mean IOP decreased by 43.1% and 42.6% at postoperative month 6 and 12. The overall rate of complete and qualified success at 6 months and one year was 79.6% / 7.4% and 74% / 7.4% respectively. Bleb failure was observed in 12.9% of the patients at 6 months and 18.5% at one year. Bleb fibrosis was managed by surgical revision using a biodegradable collagen matrix (Ologen 12 x 1 mm, Aeon Astron Corporation, Taipei, Taiwan) and MMC 0.2 mg/ml applied with sponges for 2 minutes (dose 11–17 μg [5]).

### Qualitative AS-OCT bleb analysis

Bleb wall´s reflectivity increased with time due to the decrease of both the multilayered separation of the conjunctiva and Tenon´s layer and the presence of microcysts (Table 2). The most frequent type of EFC at 6 months and 1 year was the “grade 3” type. The grade 4 (“GDD” or “zeppelin” type) was higher at 1 year compared to the first week (12% vs. 0%).

**Table 2:** Qualitative assessment of the bleb wall´s reflectivity and type of episcleral fluid cavity.

**Multilayered stroma**	*Very Low (%)*			*Low (%)*		*Medium (%)*			*High (%)*	
*1 week*	40			28		32			0	
*1 month*	27			41		24			9	
*6 months*	2			28		48			22	
*1 year*	4			10		48			38	
**Microcysts**		*None / Few (%)*					*Many (%)*			
*1 week*		56					44			
*1 month*		62					38			
*6 months*		85					15			
*1 year*		92					8			
**Episcleral fluid cavity**	*Grade 0 (%)*		*Grade1 (%)*		*Grade 2 (%)*			*Grade 3 (%)*		*Grade 4 (%)*
*1 week*	8		40		40			12		0
*1 month*	3		24		45			21		6
*6 months*	4		26		13			50		7
*1 year*	6		10		22			49		12

Results of outcome parameters of the bleb´s morphology in the first year. Percentage of patients per degree of reflectivity of the bleb wall (multilayered stroma and microcysts) and type of episcleral fluid cavity (see Fig 1).

### Quantitative AS-OCT bleb analysis

The bleb wall´s thickness and the total height of the bleb, as well as the width and total filtering surface of the episcleral fluid cavity showed a significant increase from the first month to the first postoperative year. The flow through PMS and the hydraulic conductivity of the bleb wall remained stable from 6 months to one year (7.1 ± 1.9 to 7.1 ± 1.6 (μL/min), p = 0.04; 0.04 ± 0.06, 0.03 ± 0.06 (μL/min)/mm2/mmHg, P = 0.27 Table 3.

**Table 3:** Quantitative AS-OCT bleb analysis.

	1 month	6 months	P Value	1 year	P Value
Bleb wall´s thickness (μm, ± SD)	556.5 ± 217.8	636.2 ± 222.6	0.05	650.8 ± 268.6	0.04
EFC Height (μm, ± SD)	560.7± 433.9	543.8 ± 275.2	0.41	593.04± 322.04	0.34
EFC Width (μm, ± SD)	3559.05 ± 1775.3	4548.6 ± 1872.7	0.01	4984.8 ± 1841.7	<0.001
EFC surface (mm^2^, ± SD)	26.2± 26.2	36.6 ± 28.06	0.04	45.6 ± 28.3	0.001
Bleb´s total height (μm, ± SD)	703.4 ± 625.3	1132.7 ± 389.3	<0.001	1232 ± 384.2	<0.001

Quantitative bleb measurement´s changes over the course of the study (1 month, 6 months and 1 year). EFC: Episcleral fluid cavity; SD: Standard deviation.

### Bivariate analysis

No significant correlations could be found between age, gender, pseudoexfoliation, combined surgery, central corneal thickness, baseline mean number of glaucoma medications, MD and VFI with surgical success and mean IOP at 6 months and 1 year. The percentage of patients that underwent surgical revision among those who were on 1 glaucoma medication prior to surgery was 0% vs. 20% among those who were on 2 or more glaucoma medications.

### Bleb variables and IOP

One year after surgery, thicker bleb walls were less reflective, (mean BWT, 937.1 ± 74.1 μm in the low reflectivity group vs. 499.2 ± 50.1 μm in the high reflectivity group, p<0.0001). The bivariate analysis between IOP and bleb wall´s thickness showed a negative and significant correlation (r = -0.3, p = 0.03), while the mean IOP was significantly higher in the group with higher reflectivity of the bleb wall (13.3 ± 0.5 vs. 10.9 ± 0.4 mmHg, p = 0.005). The total filtering surface and the IOP showed a negative and significant correlation at 6 months (r = -0.3, p = 0.01) and one year (r = -0.4, p = 0.02). At 6 months and one year, the mean IOP was significantly lower in patients with EFCs grade 2–4 compared to 0–1 (10.8 ± 0.3 vs. 14.4 ± 1.2 mmHg, p<0.001 at 6 months, 11.3 ± 0.3 vs. 14.3 ± 0.5 mmHg, p = 0.001 at 1 year). One year after surgery, blebs grade = 0 showed higher IOPs than the other bleb types (16 ± 0.5 mmHg vs. 11.6 ± 0.3 mmHg (p = 0.001). Blebs that grade 0–1 at month 1 showed significantly higher mean IOPs at one year (14 ± 1.2 mmHg) than blebs grade 2–3 (11.6 ± 0.6 mmHg), p = 0.04. Blebs with higher filtering surfaces showed lower hydraulic conductivities (r = -0.5, p < 0.0001). Table 4 shows the results from the quantitative bleb measurements per groups of surgical success or failure. The blebs in the group of complete surgical success showed thicker bleb walls, taller blebs, wider filtering surfaces and a lower HC of the bleb wall.

**Table 4:** Quantitative bleb measurements per group of surgical success or failure one year after surgery.

	Complete success (n = 40)	Qualified success (n = 4)	Failure (n = 10)	P value
Bleb wall´s thickness (μm ± SD)	718.9 ± 258.5	314 ± 123.1	495.8 ± 167.7	<0.01
EFC horizontal diameter (μm ± SD)	5435.9 ± 1513.5	3549.7 ± 1901.2	3356.2 ± 2333.3	<0.01
EFC height* (μm ± SD)	627.6 ± 314	679.5 ± 505.9	356 ± 123.5	0.02
EFC total filtering surface (mm^2^ ± SD)	51.4 ± 26.2	26.1 ± 27.8	25.4 ± 28.9	0.02
Total bleb height (μm ± SD)	1346.5 ± 332.8	993.5 ± 410.8	807.3 ± 256.3	<0.01
Flow (Q) (μL/min ± SD)	6.6 ± 1.3	7.7 ± 1.5	9.1 ± 1.1	<0.01
HC (μL/min)/mm^2^/mmHg ± SD)	0.01 ± 0.01	0.04 ± 0.3	0.1 ± 0.1	<0.01

The group of complete surgical success showed thicker bleb walls, wider filtering areas and larger vertical diameters of the blebs. EFC: Episcleral fluid cavity; HC: Hydraulic conductivity; SD: Standard deviation; Total bleb height: Bleb wall´s thickness + EFC height.

### Multivariate logistic regression (LR) analysis

A higher baseline IOP and a lower IOP at 1 year increased the probability of complete surgical success (ODDs ratio = 1.68, AUC of the ROC curve > 0.95) Fig 3.

## Discussion

The AS-OCT analysis of the bleb morphology in patients who had undergone PMS implantation with adjunctive MMC 0.2 mg/ml showed specific quantitative and qualitative features of the wall and EFC, most likely related to the design, composition, restriction to flow and IOP curve reported for this device [3, 6]. One year after surgery, successful blebs exhibited either a “TB-like” bleb, with a moderate filtering surface and a thick-low reflective bleb wall, or a large filtering surface along with a thinner and more reflective “capsule-like” bleb wall. Taller blebs and larger episcleral fluid cavities were two of the main features of the long-term, well-functioning bleb. In contrast, the complete absence or scarcity of fluid below Tenon´s capsule was a sign of bleb failure. AS-OCT has been shown not only to be a useful tool to evaluate bleb function in the long term but also to be able to predict the probability of bleb success or failure from the early postoperative period. The early presence of fluid spreading out through the tissues (fluid cavity and multilayered Tenon-conjunctiva) was a predictive factor of bleb success one year after surgery. Finally, a higher preoperative IOP was found to increase the probability of complete and qualified surgical success.

**Fig 4:**
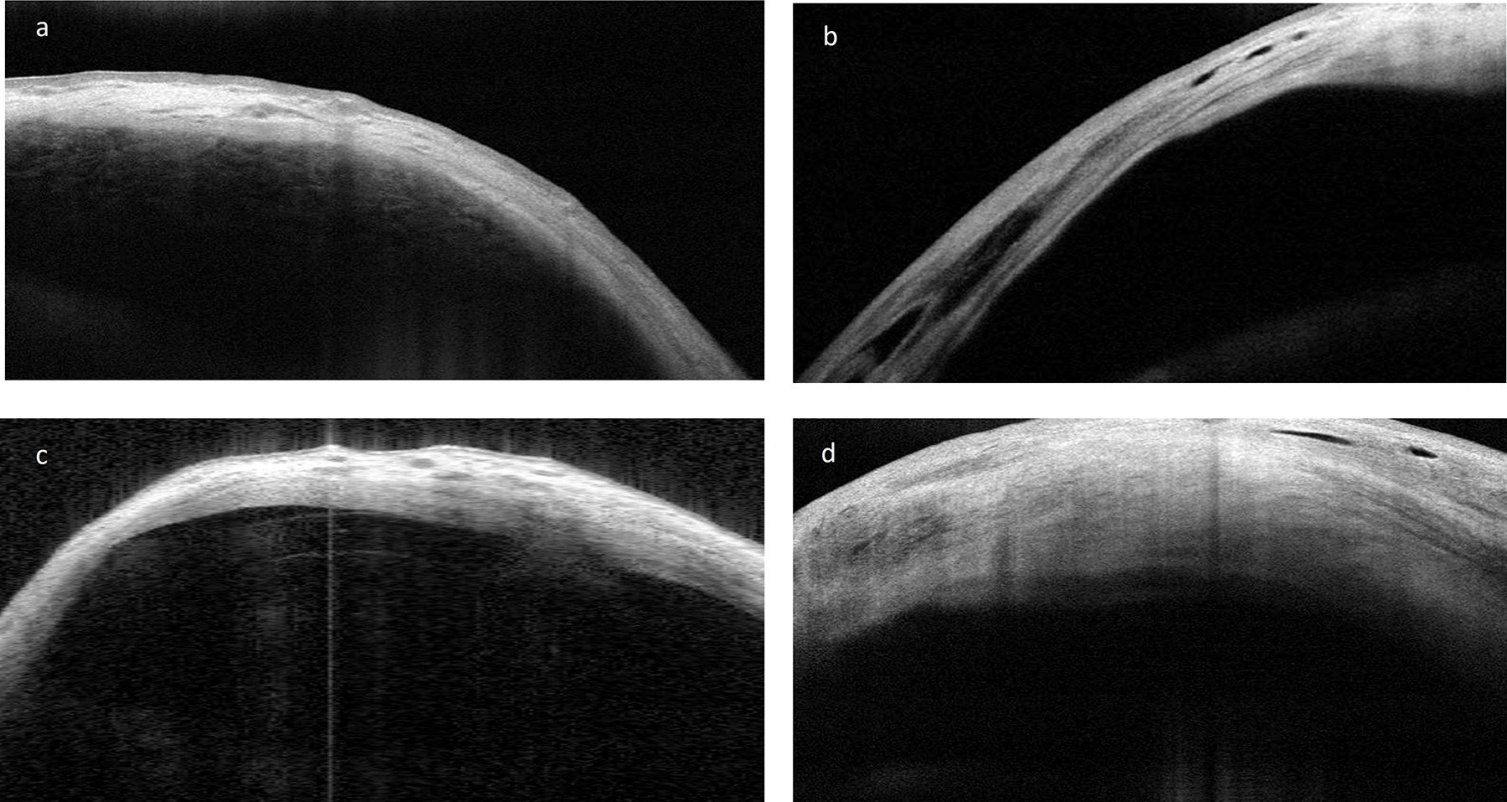
AS-OCT images from different PMS blebs and the capsule of an Ahmed valve. **a**, PMS bleb grade 4 + Ologen. **b**, PMS bleb grade 4 without Ologen due to increased hydrostatic pressure. **c**, Bleb capsule from a functioning Ahmed Valve. d) PMS bleb with Ologen that developed a thicker wall due to a shorter EFC.

The thickness of the bleb wall increased linearly over the course of the study, showing a negative correlation with the IOP (thicker walls, lower IOPs), comparable to previous reports about TB [11]. Unlike TB, the reflectivity of the bleb wall showed a trend to increase with time due to a progressive reduction of microcysts and multilayers, probably as a response to the hydrostatic pressure and geometry of the EFC. The presence of microcysts has been reported to be a biomarker of bleb success after TB [2, 11, 12] but, according to the results of the current study, not after PMS. The mean thickness of the wall appeared to be halfway between that with trabeculectomy and tube shunts (TB, 827 ± 50 μm [11]; PMS, 718.9 ± 258.5 μm, AGV, 660 ± 190 μm [13], while the reflectivity was apparently higher than that of functioning trabeculectomies [13, 14] and closer to that of the functioning capsule of the Ahmed Glaucoma Valve (AGV), suggesting a possible hybrid mechanism of action Figs 1 and 4.

A combination of different factors explains the variety of functioning blebs observed with the PMS (Fig 1). These include the specific fluid dynamics of the tube [3], the extremely hydrophobic material (SIBS) associated with a low inflammatory response [6, 7], the lack of a plate to modify the collagen deposition and orientation [14], the concentration of MMC [5], the ab-externo approach that decreases the resistance to flow and facilitates the bleb forming process [15], and the inflammatory response of the conjunctiva due to preoperative factors (thickness, reflectivity [16] and vascularization [17]). Regarding fluid dynamics, the resistance to flow through the PMS (R = 1.3 mmHg/μL/min) [3], is an invariable fluid property inherent to the design that determines the postoperative IOP reduction curve and the bleb´s internal volume and pressure. Previous studies have reported that valved drainage shunts usually show an initial good control of the IOP, frequently followed by a large rise called the “hypertensive phase” [18], which is less common after non-valved drainage devices. The IOP rise increases the tension on the capsule [19], thinning and increasing the reflectivity of the bleb wall. Thereafter, if the IOP decreases due to the use of AH suppressants or needling, the capsule becomes thicker and less reflective, increasing surgical success [20]. The PMS steadily reduces the IOP during the first months after surgery [21, 22] thanks to its fixed resistance to flow (AGVs have shown variations in their opening and closing pressures) [23], showing a linear increase in reflectivity instead of a U-shaped curve, as described by Jung and coauthors with the AGV when the capsule thins as a result of the IOP rise one month after surgery [24]. Therefore, the analysis of the bleb morphology of the PMS suggests that this device reduces the risk of the hypertensive phase.

Baseline demographics and clinical factors were analyzed as possible risk factors for surgical failure. Only a higher baseline IOP increased the probability of success in the multivariate analysis, probably due to a higher presence of aqueous that separates the tissues and increases the area of filtration. Gedde et al., in a recent publication about the treatment outcomes in the Primary Tube vs. Trabeculectomy study after 5 years of follow up reported similar results with the 350 mm^2^- Baerveldt implant. Only lower preoperative IOP was significantly associated with treatment failure in univariable and multivariable analyses [25]. A risk factor analysis from pooled data from the Ahmed Baerveldt Comparison Study, Ahmed Versus Baerveldt Study, and the tube group of the TVT Study also identified lower preoperative IOP as a significant predictor of tube shunt failure [26]. Combined and non-combined surgery showed comparable rates of surgical success and failure. Even though a higher concentration of inflammatory factors in the aqueous humor from cataract surgery could potentially influence the wound healing process of the bleb, the results from the current study did not reflect this possibility.

The absence of a plate at the end of the tube might seem like a step backward in history. Epstein back in 1958 developed probably the first tube-shunt, a tube without an end-plate that was unfortunately doomed to fibrosis [27]. Fortunately, augmented PMS surgery (MMC 0.02%) has been shown to create long-term functioning blebs, in some cases morphologically similar to tube-shunts located closer to the limbus, in other cases similar to trabeculectomy but further from the limbus (Fig 4).

According to our results, the size of the PMS bleb is comparable to TB (0.5 mm vs 0.49 mm vertical, 4.8 mm vs. 3.02 mm horizontal) [11], but is not comparable to the filtering surface of the Ahmed valve (184 mm^2^ vs. 44.7 mm^2^ PMS). In that respect, the PMS bleb would resemble TB more than tube-shunts, showing both of them a higher hydraulic conductivity of the bleb wall to compensate for the reduction of the filtering surface. In the case of PMS, (there is no data about the HC of TB), the HC is 2 orders of magnitude greater through the PMS bleb wall than through the Baerveldt capsule (0.02 vs. 0.0003 (μL/min)/mm^2^/mmHg) [14] and comparable to the HC reported by Wilcox in rabbits implanted with cylindrical devices very similar conceptually to the PMS [28]. Wilcox suggested that the HC was a biomarker of bleb success, in our opinion, the HC is just a physical property of the filtering membrane that regulates the outflow. The HC increases while the filtering surface decreases and vice versa. Interestingly, the PMS blebs grade 4 (“zeppelin” type), showed a compensatory reduction of the HC due to their large filtering surface and were morphologically reminiscent of the tube-shunt´s capsules.

One of the limitations of this study is the concentration of MMC used in this group of patients (0.2 mg/ml). Even though there is still not enough clinical evidence to recommend the use of a higher concentration of MMC, some studies have shown a trend toward a higher probability of surgical success with 0.4 mg/ml [22].

In conclusion, we have demonstrated that PMS works through a hybrid mechanism of action between trabeculectomy and tube-shunts that opens up the possibility to reconsider its actual position in the glaucoma filtering surgery spectrum, too close to trabeculectomy. Further clinical investigations about the safety and efficacy of this device compared to traditional tube-shunts, will elucidate its real utility and cost-effectiveness, since recently published studies have shown opposite cost-effectiveness results of Preserflo vs. trabeculectomy in the US Medicare system and the UK National Health Service [29, 30].
